# The Role of Functional Excipients in Solid Oral Dosage Forms to Overcome Poor Drug Dissolution and Bioavailability

**DOI:** 10.3390/pharmaceutics12050393

**Published:** 2020-04-25

**Authors:** Jannes van der Merwe, Jan Steenekamp, Dewald Steyn, Josias Hamman

**Affiliations:** Centre of Excellence for Pharmaceutical Sciences (Pharmacen™), North-West University, Private Bag X6001, Potchefstroom 2520, South Africa; 22111247@nwu.ac.za (J.v.d.M.); jan.steenekamp@nwu.ac.za (J.S.); dewald.steyn@nwu.ac.za (D.S.)

**Keywords:** solubility, bioavailability, excipients, dissolution

## Abstract

Many active pharmaceutical ingredients (APIs) exhibit poor solubility and low dissolution rates in aqueous environments such as the luminal fluids of the gastrointestinal tract. The oral bioavailability of these compounds is usually very low as a result of their poor solubility properties. In order to improve the bioavailability of these poorly soluble drugs, formulation strategies have been applied as a means to improve their aqueous solubility and dissolution rates. With respect to formulation approaches, excipients can be incorporated in the formulation to assist in the dissolution process of the drug, or specialized dosage forms can be formulated that improve dissolution rate through various mechanisms. This paper provides an overview of selected excipients (e.g., alkalinizing agents, surfactants and sugars) that can be used in formulations to increase the dissolution rate as well as specialized dosage forms such as self-emulsifying delivery systems and formulation techniques such as inclusion complexes and solid dispersions. These formulation approaches are discussed with available examples with specific reference to positive outcomes in terms of drug solubility and bioavailability enhancement.

## 1. Introduction

Pharmacologically active compounds or drugs (also referred to as active pharmaceutical ingredients; APIs) are usually not administered to patients on their own as single compounds, but are formulated into carefully designed dosage forms. Pharmaceutical dosage forms provide a platform for repeatable accurate dosing, quality, efficacy, safety, stability as well as high patient acceptance and compliance [1]. Initially, dosage forms were made by simply adding pharmacologically inert substances (also referred to as excipients) to the API to make up the required volume of an acceptable dosing unit. However, progress in pharmaceutical technology has led to the selection and production of excipients that fulfil specific functions, beyond just making up volume, such as assisting in production of the dosage form and optimizing drug delivery from novel dosage forms. In fact, the functions of excipients in dosage forms are related to all the different aspects of the final product including its manufacturability, the stability of the API, dose uniformity, effective delivery of the API to the systemic circulation after administration as well as acceptable organoleptic properties for maximum compliance by the patient [2].

Pharmaceutical excipients are usually included in dosage forms in larger quantities than the API and can make up to about 90% of the total mass/volume of medicinal products [2,3]. The International Pharmaceutical Excipient Council (IPEC) classified pharmaceutical excipients based on safety data into two classes namely ‘new chemical excipients’ and ‘established excipients’. The latter class is sub-divided into the following sub-classes: ‘existing chemical excipients’, ‘existing chemical excipients—first administration to humans’ and ‘new modifications or combinations of existing excipients’. These different classes of pharmaceutical excipients have different requirements in terms of safety evaluation [1]. Awareness of the importance of excipient quality and safety, especially in pediatric patients, was intensified by the death of 84 children in 2008 due to inclusion of glycerin contaminated with diethylene glycol in teething powders [4]. Consequently, updates on regulatory requirements for pharmaceutical excipients are continuously introduced worldwide to ensure the safety of patients [5].

The excipient class ‘new chemical excipients’ can be sub-divided into ‘modified excipients’ (i.e., existing excipients that are modified with respect to purity and/or physical properties such as particle size), ‘co-processed excipients’ (i.e., two or more existing excipients which are formulated into a new excipient with physical properties that cannot be obtained by a simple physical mixture and is produced through processes such as spray drying) and ‘novel excipients’ (i.e., new chemical entities used for the first time in a drug product which may include known excipients that are chemically modified) [6].

With the progress in the development of novel drug delivery systems, more sophisticated excipients are needed to impart certain properties into the final product. The term ‘multifunctional excipients’ refer to excipients (which can be co-processed for example) that fulfil multiple roles in a dosage form or drug delivery system, for example a direct-compressible filler material that also functions as a binder and/or disintegrant. The term ‘high functionality excipients’ refers to a single excipient that provides additional functions to innovative drug delivery systems to improve the overall performance of the product with significant economic benefits. An example of a high functionality excipient is one that can provide better flow, act as a disintegrant and simultaneously allow a higher drug loading in the dosage form due to its high compressibility [7].

It is estimated that approximately 40% of current medication dispensed to patients in pharmacies exhibit relatively poor aqueous solubility properties. Furthermore, approximately 90% of newly discovered therapeutic compounds, which are in the development phase, have also been reported to exhibit poor aqueous solubility. For these drugs, functional excipients are needed to assist in overcoming their poor physico-chemical properties. Specialty excipients are used to produce dosage forms that can reduce the number of doses by modifying the rate of drug release or improve drug delivery by targeting drug release in a specific region in the gastrointestinal tract where drug absorption is the highest. Functional excipients are also used to re-formulate existing drugs in order to produce more effective products that are more cost-effective [6,8].

Aqueous solubility and membrane permeability were identified as the two key factors that influence the bioavailability of a drug as outlined in the Biopharmaceutics Classification System (BCS). As mentioned before, many promising new drugs exhibit poor solubility, and some also exhibit poor membrane permeability [9]. Generally, a drug that exhibits an aqueous solubility lower than 0.1 mg per mL is likely to experience limited bioavailability, and its rate of absorption will be governed by the dissolution rate [10].

Poor aqueous solubility, and consequently also poor dissolution rate, is a major challenge specifically in the systemic delivery of orally administered BCS class II and IV drugs. Physico-chemical techniques that have been employed to improve the solubility of these drugs include formation of pro-drugs, formation of salts, co-precipitation, solvent evaporation and size reduction (or micronisation). Formulation strategies that have been investigated for the same purpose include melt extrusion/granulation, formation of solid dispersions and formation of inclusion complexes. Furthermore, excipients such as surfactants, polymers, super-disintegrants and multifunctional fillers have been included in dosage forms to increase the apparent solubility of drugs [11,12].

**Figure 1:**
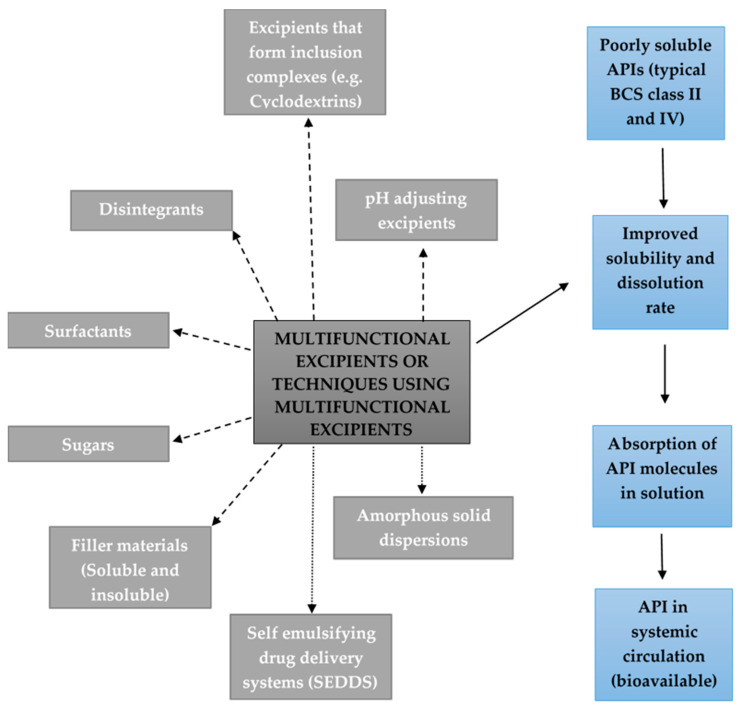
Different classes of functional excipients and techniques using functional excipients that influence either the solubility/dissolution and/or membrane permeation/absorption and consequently the bioavailability of poorly soluble active pharmaceutical ingredients (APIs).

Figure 1 is a schematic representation of different classes of functional excipients and techniques using functional excipients that improve either the solubility/dissolution and/or membrane permeation/absorption and thereby also the bioavailability of poorly soluble APIs.

## 2. Excipients Added to Solid Oral Dosage Forms to Improve Drug Solubility and/or Dissolution

### 2.1. Excipients that Form Inclusion Complexes with Drug Molecules

The term ‘complex formation’ or ‘complexation’ refers to the binding of two or more individual molecules to form a combined chemical product, which act as a single chemical unit. One of the most frequently employed complexation reactions in pharmaceutical sciences is complexation of drug molecules with cyclodextrin molecules. Cyclodextrin is developed through an enzymatic reaction with starch to form a crystalline and non-hygroscopic cone-shaped molecule that has ideal properties for complexation with certain API molecules. Cyclodextrin molecules form inclusion complexes with hydrophobic, non-polar molecules by accommodating them in the hydrophobic cavity of the cone [13,14].

Cyclodextrins (CDs) are characterized by a toroidal shape with a lipophilic center and a hydrophilic outer surface. There are three natural cyclodextrins, namely α-cyclodextrin (αCD), β-cyclodextrin (βCD) and *γ*-cyclodextrin (*γ*CD), which have six, seven and eight glucose units, respectively. The most frequent application of forming inclusion complexes between cyclodextrin and an API is to increase the apparent aqueous solubility of the API (specifically those belonging to BSC class II and IV). Other applications of complex formation include improvement of drug stability, minimization of adverse drug effects or side-effects and improvement of organoleptic properties such as taste and smell [14,15,16,17]. It is important to note, however, that not all inclusion complex formations always provide an increase in API solubility and/or absorption.

Cyclodextrins have been widely employed as solubility and dissolution modifying excipients in various oral dosage forms. These dosage forms include conventional immediate release tablets, orally disintegrating tablets, effervescent tablets, and modified release dosage forms including slow release or sustained release drug delivery systems [18,19,20]. Cyclodextrins are often preferred over organic solvents as a means to enhance solubility and dissolution due to their safety and because they are well tolerated. In addition, advances in genetic engineering, technology and process innovations led to the production of αCD, βCD as well as γCD as pharmaceutical excipients on economically and commercially acceptable scales. However, due to the relatively low solubility as well as nephrotoxicity of βCD, it is not suitable for parenteral administration. Consequently, more soluble and less toxic cyclodextrin derivatives have been developed, which include hydroxypropyl-β-cyclodextrin (HP-β-CD) and sulfobutylether-β-cyclodextrin (SBE-β-CD) [16].

Examples of the beneficial effects of cyclodextrins as functional excipients on APIs are highlighted by Conceição et al. [14], which include the increased solubility of carbamazepine by means of complexation with HP-β-CD. The effect of complexation of carbamazepine with HP-β-CD is further illustrated by a study conducted by Kou et al. [17]. A complex of HP-β-CD with carbamazepine was prepared in the presence of 0.1% hydroxypropyl methyl cellulose (HPMC). The formed complex had increased the solubility of carbamazepine up to 95 times when compared to the drug alone. Furthermore, the complexation of carbamazepine with HP-β-CD rendered a 1.5-fold increase in the bioavailability of carbamazepine in beagle dogs when compared with an immediate-release commercially available carbamazepine tablet. The HP-β-CD-containing formulation rendered a maximum plasma concentration (C_max_) of 4951.04 ± 1585.21 ng/mL and area under the curve (AUC_0–∞_) of 8597.85 ± 2786.18 ng·h/mL in comparison to a C_max_ of 3577.99 ± 1444.90 ng/mL and AUC_0–∞_ of 6000.65 ± 2227.61 ng·h/mL for the commercially available formulation. The improvement in bioavailability was attributed to an increase in dissolution rate of the HP-β-cyclodextrin complex containing formulation [17].

In a study by Desai et al. [21], orally disintegrating tablets (ODTs) were prepared containing an inclusion complex between eslicarbazepine and β-CD, which was prepared by using a solid dispersion technique. The ODT formulation exhibited 100% dissolution within 60 min compared to 72% dissolution for a commercially available tablet formulation. In an in vivo study in rabbits, the ODT formulation exhibited an improved bioavailability in comparison to the commercially available formulation as characterized by a T_max_ of 2 h, C_max_ of 6661.34 ng/mL and AUC_0–∞_ of 49,887.9 ng/mL·h in comparison to a T_max_ of 4 h, C_max_ of 2534.39 ng/mL and AUC_0–∞_ of 23,684.7 ng/mL·h for the commercial formulation [21].

### 2.2. Disintegrants

Solid oral dosage forms, regardless of their drug release mechanism, have to undergo a disintegration process. Inclusion of a disintegrant in solid oral dosage forms usually promotes faster and/or more extensive dissolution in comparison to a formulation that does not contain a disintegrant. A disintegrant is therefore an essential component in solid oral dosage forms to ensure maximum pharmaceutical availability and, consequently, also acceptable drug bioavailability [21,22].

Mechanisms through which disintegrants break dosage forms up into smaller parts include swelling, heat generation due to increased interaction between particles, exothermic wicking action, particle repulsion and recovery of particle deformation. The swelling mechanism entails that the particles of the disintegrant swell and expand when exposed to moisture. This will cause the neighboring particles to be pushed away causing expansion of the dosage form leading to its disintegration. It is important to ensure sufficient voids in the dosage form when formulating with a swelling disintegrant to allow enough moisture to enter into the dosage form to initiate the swelling process [23].

In a comparative study between jackfruit starch and croscarmellose sodium done by Suryadevara et al. [24], the excipients were investigated as superdisintegrants in the formulation of irbesartan (BCS class II drug) containing fast disintegrating tablets via wet granulation. Irbesartan and other excipient concentrations were kept constant with the only variables being the jackfruit starch and croscarmellose sodium concentration. These two disintegrants were investigated at 2.5%, 5% and 10% w/w concentrations in the tablets. Irbesartan oral dosage forms provided a delayed and prolonged pharmacological action due to its poor solubility in intestinal fluids. The need for a fast hypotensive action exists for this drug, and a fast disintegrating tablet providing high concentrations of the drug in the gastro-intestinal tract rapidly may fulfill this need. The tablet formulation containing 5% w/w jackfruit starch as superdisintegrant delivered an extent of 97.5% dissolution compared to the pure irbesartan formulation, which achieved only 43.4% dissolution after 60 min. The inclusion of jackfruit starch resulted in a 2.25-fold increase in the extent of dissolution of irbesartan and a 1.17-fold higher dissolution extent than the commercially available irbesartan product. The formulation containing croscarmellose sodium as superdisintegrant rendered an extent of dissolution of 97.9%. This dissolution rate is 2.26-fold higher than the pure irbesartan formulation and 1.18-fold higher than the product which is currently on the market. The results of this study clearly indicated that the inclusion of a superdisintegrant provided the required fast disintegrating action allowing a larger surface area for contact with the gastro-intestinal fluids at an earlier time point, which in turn provided a higher concentration of irbesartan available for absorption [24].

In another study conducted by Husseiny et al. [25], valsartan was formulated into a fast disintegrating tablet using sodium starch glycolate and crospovidone in different concentrations. According to recent research, valsartan proved to be a promising candidate for the treatment of pediatric hypertension, especially in patients suffering from either diabetes or renal impairment [26]. In the results obtained from this study, it was shown that all tablet formulations containing the highest percentage of superdisintegrant (5% *w/w*) could disintegrate within 2 min. The dissolution results indicated that both sodium starch glycolate (5% *w/w*) and crospovidone (5% *w/w*) formulations exhibited the highest percentage dissolution and also the fastest initial dissolution rate. In vivo antihypertensive activity results of the tablet formulations were in agreement with the disintegration and dissolution results, indicating that the tablet formulations containing 5% *w/w* sodium starch glycolate and 5% *w/w* crospovidone exhibited the best blood pressure lowering results. The tablet formulations lowered the arterial blood pressure by 60 and 20 mm Hg, respectively, for the formulation containing 5% *w/w* crospovidone and 5% *w/w* sodium starch glycolate within the first hour after oral administration. The reference oral suspension formulation only managed to lower the arterial blood pressure by 10 mm Hg throughout the 3 h duration of the in vivo evaluation. The fast disintegrating tablet formulations exhibited an antihypertensive effect after only 15 min post administration, whereas the oral suspension formulation exhibited the first sign of antihypertensive action 60 min after oral administration. The early disintegration and release of active ingredient from the fast disintegrating tablet formulations contributed to the early onset of action and was most likely mediated by faster and more efficient absorption of valsartan [25].

Disintegration of a solid oral dosage form and the dissolution of the active ingredient after the disintegration process is known to be crucial and can be the rate-limiting step for drug absorption. Inclusion of disintegrants in solid oral dosage forms have been proven to increase the disintegration rate of solid oral dosage forms and consequently increase the percentage dissolution, bioavailability and the pharmacological effect of certain active ingredients.

### 2.3. pH Adjusting Excipients

The pH of the boundary layer (or stagnant diffusion layer) on the surface of solid drug particles is of special importance for the dissolution of weak acidic and basic drugs [27]. It is possible to improve the dissolution rate of weak acidic and basic drugs by including pH-adjusting excipients in a formulation that is capable of changing the pH of the stagnant diffusion layer or micro-environment. pH-adjusting excipients are mixed with the other ingredients of a solid oral dosage form prior to being compressed into tablets or filled into capsule shells. When disintegration of the dosage form takes place, these excipients dissolve in the fluids of the gastrointestinal tract. These excipients then cause a pH adjustment of the stagnant diffusion layer due to provision of H^+^ or OH^−^ ions (depending on whether decreasing or increasing of pH is desired) that favors the dissolution of the active ingredient by forcing its molecules to exist more in the ionized state [28,29,30].

Examples of pH-adjusting excipients that can be used to improve the dissolution rate of weak basic drugs include organic acids (e.g., citric acid, tartaric acid and carbonic acid). After disintegration of a solid dosage form, the organic acid dissolves in the surrounding gastric juices and lowers the pH of the stagnant diffusion layer surrounding each drug particle, creating a desirable environment for the dissolution and consequently the absorption of alkaline drugs. Acidic drugs, on the other hand, would exhibit an improved solubility as well as dissolution rate in an alkaline micro-environment since they will exist more in the ionized state [28,29,30].

In a study investigating the effect of sodium bicarbonate (NaHCO_3_) and calcium carbonate (CaCO_3_) on the pharmacokinetics of paracetamol after oral administration to human subjects, it was shown that the inclusion of NaHCO_3_ (630 mg) in tablets resulted in a faster absorption of paracetamol as indicated by a lower T_max_ value and higher C_max_ value. Furthermore, the inclusion of NaHCO_3_ also reduced the variability in absorption. The increase in absorption of paracetamol upon the inclusion of NaHCO_3_ in the tablet formulations was partly attributed to an increase in in vivo dissolution rate. It is, however, interesting to note that this effect was not seen with the inclusion of CaCO_3_, and the reason for this occurrence is not clear yet [31]. In another study, the combination of Na_2_CO_3_ (an alkalizing agent) and poloxamer 407 (a polymer used in the formulation of a nano-emulsifying solid dispersion formulations) with aceclofenac (a poorly water-soluble, weak acidic drug) resulted in an increase in the in vitro dissolution rate of the drug. The increase in dissolution rate was attributed to a combination of three mechanisms, namely modulation of the microenvironmental pH, changing drug crystallinity and providing a more favorable nano-emulsion-forming environment [32]. In a follow-up study, also using aceclofenac as model drug, it was shown that the inclusion of Na_2_CO_3_ in a bilayered tablet formulation resulted in an enhanced solubility and higher plasma concentration in comparison to a reference formulation in beagle dogs. Furthermore, it was illustrated that the inclusion of Na_2_CO_3_ as alkaliniser rendered improved in vivo drug safety (decreased gastrointestinal bleeding). This improved safety profile was attributed to the effects of the alkaliser on the microenvironmental pH and a reduction in the size of the drug particles [33].

### 2.4. Amorphous Solid Dispersions

The implementation of a solid dispersion as part of a dosage form is considered one of the most effective ways to improve the aqueous solubility of an API and consequently its bioavailability. A solid dispersion describes the dispersion of one or more active ingredient(s) in a carrier matrix material. The matrix consists of a polymer in most cases, and examples include PVP (polyvinyl pyrrolidone), HPMC (hydroxypropyl methylcellulose), HPMCP (hydroxypropyl methylcellulose phthalate), chitosan, CMC (carboxymethylcellulose), sodium alginate and sodium starch glycolate. A solid dispersion system contains potential energy that is available to assist in maximizing drug release. Upon reaching a specific area in the human gastro-intestinal tract that is favorable for drug dissolution and absorption due to certain favorable environmental properties, the maximum drug concentration will be released from the dispersion. Solid dispersions can be prepared using methods such as spray drying, freeze drying, co-precipitation, fusion methods, hot melt extrusion and supercritical fluid precipitation [34,35].

When composing an amorphous solid dispersion, the drug is dispersed in a polymer matrix in the amorphous state. Studies have shown that the amorphous solid dispersion of an active ingredient increases the solubility and bioavailability compared to the crystalline state of the same drug. In a study conducted by Herbrink et al. [36], it was said that amorphous drug particles have a higher Gibbs free energy in comparison to the crystalline form, which increased the solubility of the drug. The bioavailability of the marketed product, Tasigna^®^ nilotinib, is relatively low and estimated at about 30%, which is also characterized by substantial variability. A solid dispersion of nilotinib was prepared by means of spray drying. Nilotinib was molecularly dispersed in Soluplus^®^, a polyethylene glycol, polyvinyl acetate and polyvinylcaprolactame-based graft co-polymer at a ratio of 1:7. This solid dispersion exhibited a remarkable increase in the solubility of the drug. Whether the increase in solubility of this drug will render a corresponding increase in bioavailability should be investigated by follow up in vivo studies [36].

In a recent clinical study with the drug posaconazole, a solid dispersion was formulated using hot melt extrusion to disperse posaconazole in hydroxypropyl methylcellulose (HPMC) acetate succinate (HPMC-AS) matrix. Hydroxypropyl methylcellulose (HPMC) acetate succinate is a polymer showing pH-sensitive reactions in the human gastro-intestinal tract. Results obtained by Hens et al. [37] showed that dispersing posaconazole in HPMC-AS caused a supersaturation of the drug throughout the gastro-intestinal tract and an absorption profile less dependent on the gastric pH, fed or fasted state in healthy human test subjects. This study showed that formulating a delayed release formulation provided more sufficient posaconazole release in comparison to an oral solution of posaconazole. This study showed that fasted state absorption of posaconazole was improved drastically, and further absorption along the gastro-intestinal tract was also noted due to the delayed release of posaconazole [37].

In a study conducted on dutasteride (5-α reductase inhibitor), commercially available as Avodart^®^, the active ingredient was formulated as a solid dispersion. The solid dispersion was prepared using the solvent evaporation method implemented via a vacuum desiccator. Hydrophilic excipients were also included to enhance drug dissolution. The powder type dispersion of dutasteride in combination with tocopherol polyethyleneglycol-1000-succinate, sodium lauryl sulphate and microcrystalline cellulose proved to be a successful combination in enhancing its dissolution as well as its bioavailability. Tocopherol polyethyleneglycol-100-succinate is a vitamin-E derivative and was used as a solubilizing agent to enhance drug dissolution. Sodium lauryl sulphate was included as a surfactant to enhance wettability of the solid oral dosage form, and microcrystalline cellulose was included as the carrier to inhibit recrystallization of dutasteride. The carrier medium also provided a stable environment for the active ingredient and for the other excipients. The dutasteride solid dispersion proved to be successful, and a significant increase in dissolution percentage of 10–20% was noted. The percentage of bioavailability also increased when compared to the Avodart^®^ soft capsule [38,39].

Curcumin, an extract prepared from *Curcuma longa*, has enjoyed recent attention due to the pharmacological activity which this natural compound exhibited. According to recent research, curcumin provides anti-angiogenic, anti-microbial, anti-inflammatory, anti-oxidation and anti-cancer properties [40]. Curcumin can be classified in class IV of the BCS due to its hydrophobic properties and low water solubility [40]. In an attempt to improve the solubility and membrane permeability of curcumin, Huang et al. [41] prepared an amorphous solid dispersion using Chitosan^®^ oligosaccharide as a carrier matrix. This oligosaccharide provided a low viscous, hydrophilic environment and also increased the possibility of improved membrane permeability by means of opening of the tight junctions in the gastro-intestinal epithelium. The results obtained proved that the use of this oligosaccharide attributed immensely to the solubility and permeability of curcumin. Examining the results of this study, it became evident that the solubility of the amorphous solid dispersion with curcumin and the Chitosan^®^ oligosaccharide revealed solubilization results of 97.85–101.21 µg/mL compared to the 60.62 µg/mL reached for normal curcumin. The apparent permeability results across biological membranes showed a 1.72- to 4.44-fold increase compared to that of the normal curcumin. The Chitosan^®^ oligosaccharide proved to be a successful carrier matrix for preparing an amorphous solid dispersion to ensure more efficient delivery of curcumin [41].

### 2.5. Surfactants

Solubilization of the API in the gastro-intestinal fluids after oral administration is considered a fundamental step for optimal permeability and bioavailability. In addition, not all APIs and excipients used in tablet formulations possess good aqueous wettability properties, and functional excipients are needed to enhance their wettability [42].

Surfactants are included in solid oral dosage forms to reduce the interface energy barrier between the dissolution medium and the API, which will allow for more efficient wettability. Surfactants are amphiphilic substances that contain both hydrophobic (non-polar) and hydrophilic (polar) functional groups in their molecular structures [43,44].

Surfactants are able to form micelles above the critical micelle concentration (CMC) in aqueous liquids such as the fluids found in the gastro-intestinal tract. These micelles can increase the solubility of poorly water-soluble active ingredients by incorporating these molecules in the lipophilic cores of the micelles [43].

Anionic surfactants are also known as amphiphilic surfactants because they contain both polar and non-polar functional chemical groups in their structures (e.g., sodium lauryl sulphate (SLS)). Other surfactants include cationic surfactants that are commonly used as disinfectants (e.g., cetrimide), non-ionic surfactants (e.g., alkyl betaine) and amphoteric/zwitterionic surfactants (e.g., polysorbates). Surfactants most commonly used in solid oral dosage forms are SLS, poloxamers and d-α-tocopherol polyethylene glycol succinate [39,43,44,45].

It was illustrated in a study that the inclusion of a 5% *w/v* concentration of SLS increased the dissolution rate of celecoxib significantly, and within the first 20 min of the dissolution test, the dissolution occurred three times faster. Sodium lauryl sulphate has shown the ability to increase the solubility of drugs such as tramadol hydrochloride, methocarbamol, diazepam, alprazolam, gabapentin, buspirone and acetaminophen [43]. Increased solubility of the active ingredient only occurred when the CMC was reached [42,43,45].

According to the study conducted by Varma and Panchagnula [46], the addition of d-α tocopherol polyethylene glycol succinate (TPGS) to a formulation containing the anti-cancer drug paclitaxel proved to enhance the solubility and bioavailability of this drug significantly. The aqueous solubility of paclitaxel increased drastically once the CMC (0.2 mg/mL) of TPGS was reached, and it increased 38-fold when combined with 5 mg/mL TPGS. When the paclitaxel–TPGS combination was administered orally to rats, a significant increase in drug absorption was identified. The AUC of paclitaxel increased 1.5-fold upon co-administration with TPGS. The solubilization of paclitaxel led to a higher concentration of drug available at the site of absorption, leading to an overall increase in the bioavailability of paclitaxel [46].

In a study conducted by Stoyanova et al. [42], the solubilization effect of different surfactants on the anti-inflammatory drug ibuprofen was investigated. In this study, non-ionic surfactants (Tween^®^ 20, 40, 60 and 80) and anionic surfactants (sodium dodecyl sulphate and sodium lauryl ethoxy (3) sulphate) were added to separate ibuprofen solutions. A constant concentration of each surfactant was added to respective ibuprofen solutions, and the strength of the interactions of the surfactants with ibuprofen was analyzed using thermodynamic treatment. It was evident that all of the surfactants analyzed in this study revealed an increased solubility of ibuprofen through solubilization. Regarding the non-ionic surfactants, it was clear from the results of this study that an increased solubilization of ibuprofen occurred with an increased quantity of carbon ions found in the hydrophobic chains of the polysorbates (Tween^®^). The surfactants sodium dodecyl sulphate, sodium lauryl ethoxy (3) sulphate and Tween^®^ 80 revealed an increased aqueous solubility by a factor of around 200. The surfactant that showed the highest increase in solubility of ibuprofen was Tween^®^ 80, and this polysorbate was also the only one in this study containing a double bond in the hydrophobic chain [42].

### 2.6. Self-Emulsifying Drug Delivery Systems (SEDDS)

Self-emulsifying drug delivery systems (SEDDS) represent a type of lipid-based drug delivery system that emulsifies in the gastro-intestinal tract and can be divided into three groups regarding emulsion droplet size after emulsion formation in the gastro-intestinal tract. SEDDS are emulsion delivery systems consisting of droplet sizes of >300 nm, while SMEDDS (self-micoremulsifying drug delivery system) consists of droplet sizes of <250 nm and SNEDDS (self-nanoemulsifying drug delivery system) consists of droplet sizes of <100 nm. The general discussion on SEDDS in this section will also include information regarding SMEDDS and SNEDDS. SEDDS were specifically developed to improve the slow dissolution and poor oral absorption of hydrophobic and hydrophilic macromolecular drugs. SEDDS consist of an isotropic mixture of lipids/oils, surfactants, co-surfactants and co-solvents. In most formulations, the surfactant is water soluble while the co-surfactant is lipid soluble. SEDDS are commonly administered orally in the form of soft gelatine capsules or as compressed tablets. Once the slightest agitation is applied to the dosage form in the gastro-intestinal tract, a self-emulsifying effect will be initiated inside the gastro-intestinal tract after administration. The formulation of SEDDS is a relatively new field, and there are still unresolved problems regarding its manufacturing method and the optimum combination of excipients. Research is therefore currently being undertaken regarding S-SEDDS (solid self-emulsifying drug delivery system) to establish a more physicochemical stable and viable dosage form for oral administration [47,48,49].

In a recent study conducted by Chaudhary et al. [50], the lipophilic BCS class II drug nabumetone was investigated for potential formulation into SNEDDS. Initially different formulations were prepared including different concentrations of the drug, Tween^®^ 80 (surfactant), polyethylene glycol 400 (co-surfactant), capryol-90 (oil/lipid) and an aqueous phase of deionized water. The nabumetone (40% *w/w*) was dissolved in the capryol-90, thereafter the mixture of Tween^®^ 80 and polyethylene glycol 400 (PEG 400) was added to the oil phase in small controlled quantities, while adding the aqueous deionized water dropwise under continuous vortex conditions until stable SNEDDS were noticeable. According to the results obtained, one formulation was identified to have a droplet size of 55.06 nm (±5.67 nm). This formulation contained 40% *w/w* nabumetone, 22% *w/w* capryol-90 (oil), 16% *w/w* Tween^®^ 80 and 4% *w/w* PEG 400. Regarding the in vitro dissolution study results, this SNEDDS revealed a total of 95.61% drug release of which 66% was released within the first 4 h of the dissolution test. A suspension was made containing the nabumetone commercially available tablet. This suspension exhibited a 48.53% drug release, which implies that the SNEDDS formulation provided a 1.97-fold increase in drug release over a period of 4 h. The in vivo bioavailability evaluations were done in Wistar rats (weighing 200–250 g). The in vivo bioavailability results were in agreement with the dissolution results, which showed that a C_max_ value of 45.38 ng/mL could be reached with the SNEDDS formulation, while only 23.95 ng/mL was obtained for the commercial tablet suspension, revealing a 1.89-fold increase in C_max_ for the SNEDDS [50].

Another example of the successful implementation of a lipid-based drug delivery system in the form of SMEDDS was illustrated by Kang et al. [51], where simvastatin containing SMEDDS were formulated and evaluated compared to the commercial product Zocor^®^. Simvastatin possesses hydrophobic properties and exhibits poor dissolution in the human gastro-intestinal tract, resulting in a relatively low bioavailability. The different SMEDDS formulations were prepared by dissolving the simvastatin in the co-surfactant. Thereafter, the surfactant and the oil phase were mixed together. The mixture of surfactant and oil phase was added dropwise to the mixture containing simvastatin and co-surfactant, and during the mixing process, vortexing was applied to ensure adequate mixing. Five SMEDDS formulations were prepared consisting of different concentrations of capryol 90 (oil), lauroglycol 90 (oil), carbitol (co-surfactant), PEG 400 (co-surfactant), polypropylene glycol (co-surfactant), cremophor EL (surfactant) and deionized water. In vitro drug release was evaluated using the USP 2 method with a buffered medium at a pH of 6.8. In vitro evaluation was done by means of oral administration of the SMEDDS formulations to beagle dogs. Both in vivo and in vitro results of the formulation with the smallest average droplet size of 33 nm revealed much higher drug release than the commercial tablet and revealed a C_max_ of 35.35 ng/mL compared to the commercial product exhibiting a C_max_ of 18.19 ng/mL. This formulation showed a relative bioavailability percentage of 159% [51].

### 2.7. Sugars

Sugars and sugar-containing excipients have been used for many years as multifunctional excipients in directly compressed tablets as well as other solid oral dosage forms. Most frequently used sugar type excipients include direct compressible sucrose (95% sucrose and 5% sorbitol), sucrose esters, sucrose laurate (non-ionic surfactant), crystalline maltose, mannitol and glucose. Initially, sucrose-based excipients were utilized for taste masking purposes, but currently they are also utilized for additional purposes such as compressibility of tablets, hydrophilic/hydrophobic dual properties and inclusion in nano-suspensions and nanoparticles as sucrose esters [52,53].

In a study regarding the formulation of a solid dispersion containing sucrose laurate and gemfibrozil, the solid dispersion was formed via the melting technique. This study included a toxicity evaluation to prove that the sucrose-based surface active agent did not pose the same toxic effects as many other surfactants. The effect of different concentrations (1%, 5%, 10% and 15% *w/w*) of sucrose laurate was investigated on the dissolution of the drug. Dissolution results revealed that optimal gemfibrozil release occurred using sucrose laurate in concentrations of 5–10%, obtaining 100% gemfibrozil release within 10 min compared to the 30% release reached after 10 min from the gemfibrozil suspension. These results proved a significant increase in the dissolution of gemfibrozil by sucrose laurate. Evaluation of cytotoxicity of sucrose laurate on Caco-2 cells revealed that the concentrations of sucrose laurate that mediated the best gemfibrozil dissolution (5% and 10% *w/w*) were non-toxic [54].

Mannitol is manufactured via a catalytic hydrogenation process of the natural sugars glucose or fructose. This functional excipient is highly water soluble, non-hygroscopic, non-toxic and shows little sensitivity to applied heat. Mannitol is generally recognized as safe, and this excipient is frequently used in the pharmaceutical manufacturing of solid dosage forms. Mannitol consists of highly porous particles with a relatively large surface area. Incorporation of APIs with the mannitol creates a highly porous powder mixture with improved water wettability and dissolution rate. Recent studies have shown that incorporating mannitol in formulations via physical mixtures, melting techniques or kneading methods could increase the dissolution rate of the APIs significantly [55,56].

In a study conducted by Yadav et al. [57], mannitol formulated with ketoprofen by way of three different methods (i.e., melt technique, physical mixture and kneading method) revealed an increase in ketoprofen dissolution rate, which correlated directly with the concentration of mannitol added. Ketoprofen:mannitol formulations in ratios of 1:1, 1:3 and 1:5 were formulated for each of the three methods, rendering a total of nine formulations. From the results of this study it was clear that all ratios of 1:5 (ketoprofen:mannitol) exhibited the best results with respect to drug release after 30 min in a pH 1.2 medium. The three mannitol-containing formulations outperformed the pure ketoprofen suspension by at least 51% in all three preparation methods. The highest extent of dissolution after 30 min was 72.28% for ketoprofen formulated via the kneading method. The melting method and the physical mixture method resulted in drug releases of 66.34% and 63.76%, respectively. According to Yadav et al. [57], the increased dissolution results were obtained due to a higher wettability of the powder mixture and also because of a smaller particle size obtained after dispersion of the drug in the mannitol creating a higher contact surface area. The contribution of a decrease in particle size to dissolution was also confirmed by a study conducted by Nassab et al. [56], regarding the drug meloxicam, where the particle size of meloxicam was varied and dispersed in mannitol. Results in this study showed an increase in drug release with smaller size particles of meloxicam and with an increase in the concentration of mannitol. Several studies showed that mannitol can increase the dissolution rate of hydrophobic drugs via more than one mechanism [56,57], such as the attraction of water molecules into the dosage form due to better wettability of the dosage form that also causes an increase in dissolution rate of the active ingredient.

### 2.8. Soluble and Insoluble Filler Materials

A filler is an excipient normally used in solid oral dosage forms to provide sufficient mass and volume. Multifunctional fillers are now used in solid oral dosage forms to fulfill more than one function. Two major groups of pharmaceutical filler materials exist, namely aqueous soluble and aqueous insoluble fillers. Fillers for oral solid dosage forms are chosen to help reach the best possible drug release profile from the dosage form in the environment of the human gastro-intestinal tract [1,58].

MCC (microcrystalline cellulose) is one of the most frequent used filler materials in the pharmaceutical industry to produce direct compressible tablets and beads. MCC is purified cellulose that is partially depolymerized and is obtained from fibrous plant material (e.g., wood) [58,59]. In a recent study, the antioxidant quercetin was combined with MCC via a crystallization technique called fluid bed crystallization. MCC was placed in the fluid bed followed by a quercetin–acetone mixture of specific concentrations. Quercetin nanorods were then dispersed onto the surfaces of the MCC particles after dehydration. In vitro dissolution testing was conducted over 2 h in 0.1 M HCl to represent the gastric pH of 1.2. The antioxidant effects of the quercetin formulations were evaluated in vitro via utilization of a DPPH (2,2-diphenyl-1-picrylhydrazyl) scavenging assay. A decreased value of DPPH absorbance indicated an increased antioxidant effect by quercetin. The dissolution increased significantly compared to the quercetin control formulation. The top performing formulation indicated more than double the drug release (34.8%) after only 5 min compared to the quercetin control formulation (15.6%). The total drug release after 2 h was 76.2% for the top performing formulation and 56.4% for the quercetin control formulation. The in vitro antioxidant evaluation revealed a constant increase in inhibitory effect, with values being significantly higher than the quercetin control formulation at 20, 60 and 120 min. The dispersion of quercetin particles onto the surface of the MCC decreased the particle size of the drug throughout the mixture, thereby increasing the surface area available for dissolution medium contact and thus increasing the dissolution of the drug and also the antioxidant effect [60].

An example of an aqueous soluble filler that has been used extensively in the pharmaceutical industry for many years is lactose. The most common forms of lactose used in solid oral dosage form formulations are crystalline alpha-lactose monohydrate, beta-lactose (crystalline anhydrous lactose) and amorphous lactose. Uptake of the dissolution medium in a dosage form is of importance to achieve optimal dissolution and bioavailability of the active ingredient inside the dosage form. Formulating a solid oral dosage form with an aqueous soluble diluent that attracts water towards the dosage form may contribute to enhanced dissolution rate of hydrophobic/poorly aqueous soluble APIs [61].

Li et al. [62] used lactose as carrier for a nanodispersion containing bicalutamide. Bicalutamide is a BCS class II drug exhibiting poor solubility in the gastro-intestinal fluids leading to low dissolution and bioavailability. It was stated that the small particle size of this drug caused agglomeration of the powder mixtures, and it was best to keep this drug dispersed in a carrier medium. Nanodispersions were formulated using the liquid precipitation method dispersing the bicalutamide particles onto the surface of the lactose creating a large contact surface area for optimal solubility. Other formulations contained different functional excipients also formulated for enhanced dissolution purposes. All formulations containing functional excipients revealed enhanced dissolution and drug release from the nanodispersions. All formulations exhibited equal or more than 50% drug release within the first 10 min, but the dispersion formulated using lactose as carrier revealed a 94% drug release within 10 min and 99% release within a period of 2 h. An MCC–bicalutamide formulation was also formulated showing less than 50% drug release within 10 min and only 85% release after 2 h. The control bicalutamide formulation only managed 30% release after 10 min and 78% after the 2 h period. The experimental formulations were compared to that of a reference drug product on the market (Casodex^®^), which showed 60% drug release after 10 min. Not only did the lactose–bicalutamide combination exhibit faster release of the API but also a higher percentage release overall during the 2 h evaluation period. The key to the success in this study was mediated by smaller particles of a poorly aqueous soluble drug dispersed onto aqueous soluble carrier particles attracting water into the formulation [62].

A summary of excipients that have been investigated and used in dosage forms for improvement of drug solubility and dissolution rate is given in Table 1.

## 3. Conclusions

Various approaches can be followed or employed to improve the dissolution of a poorly soluble API in a solid oral dosage form ranging from incorporation of functional excipients to techniques such as preparation of solid dispersions and micronisation of the API particles. Examples of excipients that can increase an API’s solubility and dissolution rate include cyclodextrins, disintegrants, pH-adjusting excipients, natural polymers, surfactants, co-surfactants, oils/lipids and sugars.

Several studies have shown that different classes of functional excipients have a definite and beneficial effect on the solubility/dissolution and consequently the bioavailability of poorly soluble APIs. For certain excipient groups such as super disintegrants and pH-adjusting excipients, in vivo and clinical studies seem to be limited. Furthermore, most studies only used a single, poorly soluble API as representative compound. There is a need for further research to show the applicability of (multi)functional excipients to enhance the solubility/dissolution and consequently the bioavailability of a wide variety of poorly soluble APIs. It is of special interest to determine the clinical significance or value of formulation approaches on drug bioavailability and if they could result in decreasing the dose needed to reach effective blood plasma levels. Considering that approximately 40% of current medication products contain APIs that exhibit poor aqueous solubility, and approximately 90% of newly discovered therapeutic compounds in the development phase exhibit poor aqueous solubility, future research regarding the identification of solubility-enhancing excipients is of cardinal importance.

Identifying the correct formulation method and using the suitable excipient in relation to the API may result in efficient pharmacological treatment by means of administrating a lower dose, which may have a significant cost implication.

## Figures and Tables

**Table 1:** Summary of functional excipients used in different types of dosage forms to improve drug solubility and dissolution rate.

Excipient	Excipient Subdivision	Drug Example	In Vitro/In Vivo	Dosage Form Type	Reference
Cyclodextrin	β-Cyclodextrin	Eslicarbazepine	in vitro + in vivo	Orodispersable tablet (solid dispersion)	[21]
	HP(2-Hydroxypropyl)-β-Cyclodextrin	Carbamazepine, Naproxen	in vitro + in vivo	Immediate release tablet/ Enteric coated tablet	[14,17]
Disintegrants	Croscarmellose Sodium and Jackfruit starch	Irbesartan	in vitro + in vivo	Direct compressed fast disintegrating tablet	[24]
	Sodium starch glycolate and crospovidone	Valsartan	in vitro + in vivo	Direct compressed fast disintegrating tablets	[25]
pH adjusting excipients	Citric acid	Ketoconazole	in vitro + in vivo	Physical mixture (granules)	[30]
	Tartaric acid	Ketoconazole	in vitro + in vivo	Physical mixture (granules)	[30]
	Sodium Hydrogen Carbonate	Paracetamol	in vitro + in vivo	Controlled release matrix tablet	[33]
	Calcium Carbonate	Paracetamol	in vitro + in vivo	Controlled release matrix tablet	[33]
	di-Sodium Carbonate	Aceclofenac	in vitro + in vivo	Controlled release matrix tablet	[33]
	di-Sodium Carbonate	Aceclofenac	in vitro + in vivo	Nanoemulsifying GUC (Gelucire 44/14)-based solid dispersions	[32]
Solid dispersions	Tocopherol polyethyleneglycol-1000-succinate	Dutasteride	in vitro + in vivo	Physical mixture (solid dispersion)	[38]
	Polyethylene glycol, polyvinyl acetate and polyvinylcaprolactame-based graft co-polymer	Nilotinib	in vitro	Encapsulated physical mixture (spray-dried mixture)	[36]
	Hydroxypropyl methylcellulose acetate succinate	Posaconazole	in vitro + in vivo	Delayed release tablet	[37]
	Chitosan	Curcumin	in vitro + in vivo	Amorphous solid dispersion	[41]
Surfactant	Sodium lauryl sulphate	Celecoxib, Tramadol, Methocarbamol Diazepam, Alprazolam, Buspirone, Gabapentin and Acetaminophen	in vitro	Direct compressed tablet	[43]
	Tween 20, Tween 40, Tween 60, Tween 80, sodium dodecyl sulphate and sodium lauryl ethoxy (3) sulphate	Ibuprofen	in vitro	Oral solution	[42]
	D-α-tocopherol polyethylene glycol 1000 succinate	Paclitaxel	in vitro + in vivo	Oral mixture	[46]
	D-α-tocopherol polyethylene glycol 1000 succinate	Dutasteride	in vitro + in vivo	Physical mixture (solid dispersion)	[38]
SNEDDS	Capryol-90, Tween 80 and PEG-400	Nabumetone	in vitro + in vivo	Oral SNEDDS	[50]
SMEDDS	capryol 90, lauroglycol 90, carbitol, PEG 400, polypropylene glycol and cremophor EL	Simvastatin	in vitro + in vivo	Oral SMEDDS	[51]
Mucoadhesive/ Mucopenetrating polymer	Chitosan	Telmisartan	in vitro + in vivo	Oral co-crystals	[63]
	Chitosan	Carvedilol	in vitro + in vivo	Oral nanoparticles	[64]
Sugars	Sucrose laurate	Gemfibrozil	in vitro	Oral solid dispersion	[54]
	Mannitol	Ketoprofen	in vitro + in vivo	Oral co-crystals	[57]
	Mannitol	Meloxicam	in vitro + in vivo	Oral co-crystals	[56]
Soluble and insoluble fillers	MCC	Quercetin	in vitro + in vivo	Oral co-crystals	[60]
	Lactose	Bicalutamide	in vitro	Oral nanodispersion	[62]
